# The relative importance of ski resort- and weather-related characteristics when going alpine skiing: Data from a rating-based conjoint survey

**DOI:** 10.1016/j.dib.2021.107252

**Published:** 2021-06-29

**Authors:** Erik Haugom, Iveta Malasevska, Gudbrand Lien

**Affiliations:** Inland Norway University of Applied Sciences, Lillehammer, Norway

**Keywords:** Rating-based conjoint, Alpine skiing, Weather preferences, Ski resort characteristics

## Abstract

The data are related to two research articles: “The relative importance of ski resort-and weather-related characteristics when going alpine skiing” [1] and “Optimal pricing of alpine ski passes in the case of crowdedness and reduced skiing capacity” [2]. A rating-based conjoint survey experiment on active alpine skiers at a big ski area located in Inland Norway was performed in February of 2018 to collect the data and pertain to 400 respondents doing more than 7200 ratings. A total of ten versions of the same questionnaire type were used to obtain information about preferences on ski resort- and weather-related characteristics when going alpine skiing. We display the raw data organized such that they can be easily downloaded and used directly to either (1) replicate the analyses performed in the related research articles, or (2) run one's own analyses on the topic of interest. The data may also be useful to lecturers teaching students about the key concepts of survey experiments and causal modelling.

## Specifications Table

**Table:** 

Subject	Business, Management and decision sciences
Specific subject area	Tourism, Leisure and Hospitality Management
Type of data	Table in a spreadsheet.
How data were acquired	A rating based conjoint survey experiment among existing skiers at a big ski area located in Inland Norway using ten versions of the same questionnaire type. The questionnaires are provided as a supplementary file.
Data format	Raw data and calculated variables needed to replicate analyses in related research article(s).
Parameters for data collection	At least 30 observations for each of the 10 experiment groups.
Description of data collection	The data was collected in a survey conducted at a major alpine ski area in the Inland region of Norway. We used third year bachelor students at site to recruit alpine skiers and snowboarders. The ten questionnaire types were shuffled randomly such that all respondents had a 10% chance of receiving any of the ten questionnaire types.
Data source location	Institution: Hafjell Alpinsenter City/Town/Region: Øyer/Inland county Country: Norway Latitude and longitude (and GPS coordinates, if possible) for collected samples/data: 61.234381; 10.448835
Data accessibility	The data can be accessed at Mendeley Data: Haugom, Erik; Malasevska, Iveta; Lien, Gudbrand (2021), “The relative importance of ski resort- and weather-related characteristics when going alpine skiing: data from a rating-based conjoint survey”, Mendeley Data, V1, https://doi.org/10.17632/6w4tzrs3yw.1
Related research article	The data are used in two related articles: Erik Haugom, Iveta Malasevska, The relative importance of ski resort-and weather-related characteristics when going alpine skiing. *Cogent Social Sciences*, *5*(1), (2019). https://doi.org/10.1080/23311886.2019.1681246 Erik Haugom, Iveta Malasevska, Gudbrand Lien, Optimal pricing of alpine ski passes in the case of crowdedness and reduced skiing capacity. *Empirical Economics*, (2020). https://doi.org/10.1007/s00181-020-01872-w

## Value of the Data

• Data on the relative importance of ski resort- or weather-related characteristics when going alpine skiing typically stems from traditional surveys or interviews. The data offered here uses an experimental design combined with the rating-based conjoint methodology and contains information about the users self-reported likelihood of visiting the ski area under various conditions. The data is useful to managers or analysts that want to learn how to calculate the relative importance of various attributes and how to back-out price-response functions for subsequent use in pricing models with similar data.
• In addition to managers and analysts in the industry, academic researchers and lecturers may also benefit from using the data when studying user preferences with rating-based conjoint data or teaching students about this methodology in general. A possible approach for lecturers wanting to build a module in a methods course using the data and related questionnaires could be the following:
  ○ Step 1: How to development conjoint questionnaire using survey experiments.
  ○ Step 2: Data collection and preparation.
  ○ Step 3: Descriptive statistics and data visualization.
  ○ Step 4: Calculations of relative importance and part-worth functions.
  ○ Step 5: Estimating price-response functions.
  ○ Step 6: Calculations of optimal prices for various levels of key attributes.
• The data may be used to gain further insight by replicating and extending the analyses performed and reported in the related research articles. For example, it is possible to include more explanatory variables, transform some of those already used into new variables, create interaction-variables, and more. The data and survey experiment in general may also be used as a starting point when other scholars want to create their own experiments based on similar or related methodology.

## 1 Data Description

The data stem from rating-based conjoint survey experiment among existing skiers at a big ski area located in Inland Norway. We have used ten versions of the same questionnaire type in the data collection process and the questionnaires are provided as supplementary files. A skiing day experience can differ substantially for various ski resort characteristics and weather conditions. To capture a range of various likely skiing day scenarios we focused on key weather attributes (cloud cover, temperature, and wind), ski resort characteristics (slopes open, waiting time) and time period for the skiing activity. The levels the weather attributes could take were based on likely weather variations during a normal winter in the area the survey was conducted. For the ski resort characteristics the levels were based on likely variations of waiting times in the main lifts and fractions of slopes open for a typical Norwegian alpine ski area. The use of various versions of the same questionnaire allowed us to manipulate the levels of the various attributes to create unique skiing day scenarios. The details of the experimental design is described in the next section.

The data collection was carried out by Bachelor students at a major alpine ski area in the Inland region of Norway. The students were instructed to approach adult skiers either in the Gondola or in one of the many eateries. The ten questionnaire types were shuffled randomly such that each respondent the students approached would have the same chance of receiving a given version of the questionnaire. We did not formally record refusals, but the general feedback from the students performing the field work was that most of their approaches induced a positive response and a total of 350 out of 400 respondents completed all 18 ratings in the conjoint part of the questionnaire.

In Table 1 we show information about the various items included in the data table accompanying the current article. The table includes information about the values the various variables can assume and a brief description of variable type. The accompanying questionnaires provide detailed information of the variables included in Table 1.

**Table 1:** Description of data items accompanying the article.

Variable	Description/Values	Variable	Description/Values
ID	Respondent ID: [1-400]	FEMALE	DV for female [0,1]
QUESTIONAIRE_CODE	[SLOPES-1, SLOPES-2, RAIN-1, SNOW-1, SNOW-2, TEMP-1, TEMP-2, TP-1, WIND-1, WIND-2]	AGE	In years [13-71]
QUESTION	Rating question: [8,9]	HAFJELL_RESIDENCE	Residence visit [0-4]
CODE-QUESTION	Unique code-question identifier	SKIING_DAYS_TOTAL	Days [0-100]
QUE_TIME	Que time in minutes: [1,5,10]	SKIING_DAYS_HAFJELL	Days [0-100]
WEKKDAY	Weekday: [Midweek, Weekend]	SKIING_MIDWEEK	DV for midweek days [0,1]
PRICE	Day pass NOK: [250, 350, 450, 550, 650]	SKIING_WEEKEND	DV for weekend days [0,1]
PERIOD	Time period: [Regular week, Vacation]	SKIING_CHRISTMAS	DV for Christmas [0,1]
WEATHER	[Sun, rain, cloudy, snow, fog]	SKIING_EASTER	DV for Easter [0,1]
TEMPERATURE	[-20, -15, -10, -5, 0, 5]	SKIING_WINTER-VACATION	DV for winter vacation [0,1]
WIND	[No wind, gentle breeze, fresh breeze]	SKIING_REGULAR_WEEK	DV for regular week [0,1]
SLOPES_OPEN	[0.50, 0.75, 1.00]	SKIING_ALL_PERIODS	DV for all periods [0,1]
R8-HELP	Which specific alternative in Q8/9 are rated	WORK	Work category [0-4]
RATING	User rating [0 – 100]	WORK_FULL_TIME	DV for full time work [0,1]
QUE_1	DV for Queue=1 minute [0,1]	WORK_PART_TIME	DV for part time work [0,1]
QUE_5	DV for Queue=5 minutes [0,1]	WORK_UNEMPLOYED	DV for unemployed [0,1]
QUE_10	DV for Queue=10 minutes [0,1]	WORK_STUDENT	DV for student [0,1]
MIDWEEK	DV for Midweek skiing [0,1]	WORK_OTHER	DV for other work [0,1]
WEEKEND	DV for Weekend skiing [0,1]	FAMILYSTATUS	Family status category [0-4]
REGULAR_WEEK	DV for Regular week [0,1]	SINGEL	DV for single [0,1]
VACATION	DV for Vacation [0,1]	SINGEL_WCHILD	DV for single w/child [0,1]
P250	DV for price=250 [0,1]	COUPLE	DV for couple [0,1]
P350	DV for price=350 [0,1]	COUPLE_WCHILD	DV for couple w/child [0,1]
P450	DV for price=450 [0,1]	OTHER	DV for other [0,1]
P550	DV for price=550 [0,1]	DISTANCE	Distance from resort in KM
P650	DV for price=650 [0,1]	ANC_SKI_RENT	DV for ski rental [0,1]
SUN	DV for sunny weather [0,1]	ANC_RESTAURANTS	DV for restaurants [0,1]
RAIN	DV for rainy weather [0,1]	ANC_SKISCHOOL	DV for ski school [0,1]
SNOW	DV for snow [0,1]	ANC_CHILD_ACTIVITIES	DV for child activities [0,1]
CLOUD	DV for cloudy weather [0,1]	ANC_SPORT_STORE	DV for sport store [0,1]
FOG	DV for foggy weather [0,1]	GEAR	Gear used [0,2]
TEMP_-20	DV for temp=-20 [0,1]	GEAR_ALPIN_SKI	DV for alpine skis [0,1]
TEMP_-15	DV for temp=-15 [0,1]	GEAR_SNOWBOARD	DV for snowboard [0,1]
TEMP_-10	DV for temp=-10 [0,1]	GEAR_OTHER	DV for other gear [0,1]
TEMP_-5	DV for temp=-5 [0,1]	INCOME	Income category [0,6]
TEMP_0	DV for temp=0 [0,1]	INCOME_L100	DV for income < 100’ [0,1]
TEMP_P5	DV for temp=5 [0,1]	INCOME_100-300	DV 100’ < income < 300’ [0,1]
NO_WIND	DV for no wind [0,1]	INCOME_300-600	DV 300’ < income < 600’ [0,1]
GENTLE_BREEZE	DV for gentle breeze [0,1]	INCOME_600-900	DV 600’ < income < 900’ [0,1]
FRESH_BREEZE	DV for fresh breeze [0,1]	INCOME_900-1200	DV 900’ < income < 1200’ [0,1]
SLOPES_50	DV for 50% of slopes open [0,1]	INCOME_M1200	DV for income > 1200’ [0,1]
SLOPES_75	DV for 75% of slopes open [0,1]	INCOME_NOANSWER	DV for no answer income [0,1]
SLOPES_100	DV for 100% of slopes open [0,1]	SKIINTERESS	Scale [1–7]
MALE	DV for male [0,1]		

*Note:* DV = dummy variable.

## 2 Experimental Design, Materials and Methods

The data stem from a rating-based conjoint [3] survey experiment (see [4] and [5]) among existing skiers at a big ski area located in Inland Norway using ten versions of the same questionnaire type. A survey experiment is a *“…deliberate manipulation of the form or placement of items in a survey instrument, for purposes of inferring how public opinion works in the real world.”* [4] The main argument for combining the rating-based conjoint method with a survey experiment is because a skiing day can differ substantially across ski resort- and weather-related characteristics. Hence, to avoid the use of a very complex questionnaire and at the same time retain a rich information set, we manipulated the form by changing the attribute levels of some key attributes. This approach resulted in ten versions of the questionnaire as described by the variable QUESTIONNAIRE_CODE in Table 1.

The attributes that were subject to manipulation in their levels were: weather conditions (rainy, snowy, sunny, foggy, cloudy, windy), the share of ski slopes open (50%,75%,100%), waiting time in the main lifts (1 min, 5 min, 10 min), weekday (midweek, weekend), and the time period of skiing (vacation, regular week). It is important to note that all respondents rated the same attributes, but the attribute *levels* varied across the ten versions of the questionnaire as outlined in Table 1. Hence, we believe the challenges associated with data fusion, namely uncertain and conflicting values as described in [6], are not present in the current data. The number of respondents with complete evaluations of each attribute was 350 and above the minimum sample size of 150 as suggested by [7].

The respondent was given one out of ten possible questionnaires each with a unique combination of the values the experimental variables took. The values the experimental variables assume for each questionnaire type may be read directly from the data file and are also included in Table 1. A more detailed description of the variables and their corresponding values can also be found in the accompanying questionnaires. The way the manipulation is set up ensures that all combinations of the experimental items are covered.

## Ethics Statement

All respondents participating in the survey experiment provided their informed consent before participating.

## CRediT Author Statement

**Erik Haugom:** Project administration, Conceptualization, Formal Analysis, Software, Writing - original draft preparation; **Iveta Malasevska:** Methodology, Data curation, Validation, Writing - reviewing & editing; **Gudbrand Lien:** Formal Analysis, Supervision, Software, Validation, Writing - reviewing & editing.

## Declaration of Competing Interest

The authors declare that they have no known competing financial interests or personal relationships which have or could be perceived to have influenced the work reported in this article.

## Appendix Supplementary materials

Image, application 1

Image, application 2

Image, application 3

Image, application 4

Image, application 5

Image, application 6

Image, application 7

Image, application 8

Image, application 9

Image, application 10

Image, application 11

Image, application 12

Image, application 13
