# Supplementary material for: The relative importance of ski resort- and weather-related characteristics when going alpine skiing: Data from a rating-based conjoint survey
Source: Data Brief. 2021 Jun 29;37:107252. doi: 10.1016/j.dib.2021.107252 (PMC8258849; doi:10.1016/j.dib.2021.107252)
Supplement: Supplementary file 5 [file mmc5.pdf]

CODE: SNOW-1

CUSTOMER SURVEY ABOUT SKIERS' PREFERENCES AT HAFJELL SKI RESORT

This survey is a part of a research project about skiers' preferences when visiting a ski resort.
NOTE: THE QUESTIONNAIRE HAS TWO PAGES. We highly appreciate your response.

1. Gender?
   - ❑ Male
   - ❑ Female

2. Age?
   _____ years old

3. What is your place of residence?
   City/location: ____________________
   Country: ____________________

4. Where do you stay when visiting Hafjell today?
   - ❑ At home
   - ❑ My own cabin
   - ❑ Rented apartment / cabin
   - ❑ Hotel
   - ❑ Other: _________________________

5. In a typical season, approximately, how many days do you ski in a ski resort?
   ______ days

6. In a typical season, approximately, how many days do you ski in Hafjell ski resort?
   ______ days

7. When do you typically visit the ski resort? (Several options possible)

WEEKDAY:	TIME PERIOD:
❑ Mid-week (Mon-Thu)	❑ Christmas holiday
	❑ Eastern holiday
	❑ Winter-break
❑ Weekends (Fri-Sun)	❑ Regular weeks
	❑ All of the above

8. For each proposed scenario, please indicate how likely you would be to go skiing in each case, on a scale from 0 ("Would definitely **not** go skiing in that given scenario") to 100 ("Would definitely go skiing in that given scenario"). You may choose any number between 0 and 100. (For example, feel free to use numbers such as 37, 50, 92, etc.). **The given price is for a one-day ski pass in all cases!**

You shall also assume that the given scenarios below are for skiing day characteristics as given in the top of the table below. Please read this information carefully before you give your rating.

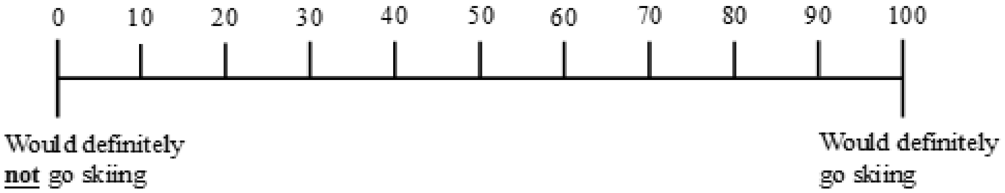

SKIING DAY CHARACTERISTICS: REGULAR WEEK (OUTSIDE ALL HOLIDAY SEASONS), AND WELL GROOMED SLOPES. WEATHER ACCORDING TO THE SYMBOLS BELOW:

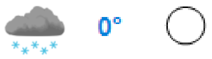

Calm,
0.0 – 0.2 m/s

PROFILE	WAITING TIME MAIN LIFTS	WEEKDAY	PRICE (ONE-DAY)	YOUR RATING
1	10 MIN	MID-WEEK (MON-THU)	250	
2	5 MIN	MID-WEEK (MON-THU)	350	
3	5 MIN	MID-WEEK (MON-THU)	450	
4	1 MIN	MID-WEEK (MON-THU)	550	
5	10 MIN	WEEKEND (FRI-SUN)	250	
6	10 MIN	WEEKEND (FRI-SUN)	350	
7	5 MIN	WEEKEND (FRI-SUN)	450	
8	5 MIN	WEEKEND (FRI-SUN)	550	
9	1 MIN	WEEKEND (FRI-SUN)	650	

9. Do the same evaluation but with these new conditions:

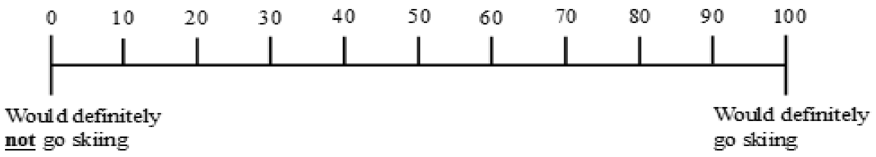

SKIING DAY CHARACTERISTICS: REGULAR WEEK (OUTSIDE ALL HOLIDAY SEASONS), AND WELL GROOMED SLOPES. **NEW WEATHER SCENARIO** ACCORDING TO THE SYMBOLS BELOW:

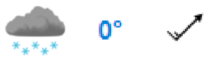

Moderate breeze,
5.5 – 7.9 m/s

PROFILE	WAITING TIME MAIN LIFTS	WEEKDAY	PRICE (ONE-DAY)	YOUR RATING
1	10 MIN	MID-WEEK (MON-THU)	250	
2	5 MIN	MID-WEEK (MON-THU)	350	
3	5 MIN	MID-WEEK (MON-THU)	450	
4	1 MIN	MID-WEEK (MON-THU)	550	
5	10 MIN	WEEKEND (FRI-SUN)	250	
6	10 MIN	WEEKEND (FRI-SUN)	350	
7	5 MIN	WEEKEND (FRI-SUN)	450	
8	5 MIN	WEEKEND (FRI-SUN)	550	
9	1 MIN	WEEKEND (FRI-SUN)	650	

BACKGROUND AND SKIING PREFERENCES

10. What is your current occupation?
    - ❑ Working full time
    - ❑ Working part time
    - ❑ Unemployed
    - ❑ Student
    - ❑ Other: ____________________

11. Family status?
    - ❑ Single
    - ❑ Single with children
    - ❑ Couple
    - ❑ Couple with children
    - ❑ Other: _____________________

12. If you are living in Norway, what is the approximate distance from your home to Hafjell Ski Resort?
    ______ KM

13. When visiting Hafjell ski resort, which of the following services do you also use? (Several options possible)
    - ❑ Ski/snowboard rental
    - ❑ Restaurants
    - ❑ Ski instruction
    - ❑ Children activities
    - ❑ Sporting good store

14. When you visit a ski resort, do you usually:
    - ❑ Go alpine skiing
    - ❑ Go snowboarding
    - ❑ Other

15. What is your household's approximate NET income?
    - ❑ Below NOK 100 000
    - ❑ NOK 100 000 – NOK 300 000
    - ❑ NOK 300 001 – NOK 600 000
    - ❑ NOK 600 001 – NOK 900 000
    - ❑ NOK 900 001 – NOK 1 200 000
    - ❑ More than NOK 1 200 000
    - ❑ Prefer not to answer

16. How interested are you in skiing in general? *Please evaluate on a scale from 1-7, where 1 = not interested at all, and 7 = very interested.*

    1 2 3 4 5 6 7

THANK YOU!
