# Supplementary material for: The relative importance of ski resort- and weather-related characteristics when going alpine skiing: Data from a rating-based conjoint survey
Source: Data Brief. 2021 Jun 29;37:107252. doi: 10.1016/j.dib.2021.107252 (PMC8258849; doi:10.1016/j.dib.2021.107252)
Supplement: Supplementary file 12 [file mmc12.pdf]

Haugom & Malasevska, *Cogent Social Sciences* (2019), 5: 1681246
https://doi.org/10.1080/23311886.2019.1681246

cogent social sciences

Received: 29 March 2019
Accepted: 13 October 2019
First Published: 18 October 2019

*Corresponding author: Erik Haugom, Inland Norway University of Applied Sciences, Postboks 400, Elverum 2418 Norway
Email: erik.haugom@inn.no

#The authors recognize the research committee at Inland Norway University of Applied Sciences for granting NOK 15.000 to collect the data. The study is also part of the research project "Innovative Pricing Approaches in the Alpine Skiing Industry" funded by the Regional Research Funds in Norway (RFF Innlandet) and Inland Norway University of Applied Sciences – The Business School.

Reviewing editor:
Ganghua Chen, Sun Yat-Sen University, Guangzhou, China

Additional information is available at the end of the article

LEISURE & TOURISM | RESEARCH ARTICLE

The relative importance of ski resort- and weather-related characteristics when going alpine skiing

Erik Haugom[1*] and Iveta Malasevska[2#]

Abstract: In this paper, we examine how alpine skiers evaluate various ski resort- and weather-related characteristics using a conjoint framework. The analysis is based on survey data from 400 existing skiers at a large ski resort in the Inland region of Norway. The results suggest that the weather of the skiing day and the price of a lift ticket are the most important attributes to alpine skiers, followed by ski lift queue time and the fraction of slopes open. The calculated part-worth functions also reveal the most desirable and most unpleasant weather conditions for alpine skiing. This insight can be used directly by ski resort managers when forming new pricing strategies.

Subjects: Sports and Leisure; Tourism, Hospitality and Events; Economics, Finance, Business & Industry

Keywords: Alpine skiing; ski resort preferences; ski resort characteristics; skiing weather; skiing temperature

ABOUT THE AUTHORS

The paper *"The relative importance of ski resort- and weather-related characteristics when going alpine skiing"* is part of the research project "Innovative Pricing Approaches in the Alpine Skiing Industry – IPAASKI" and partly funded by Regionalt Forskningsfond Innlandet (RFF), grant number 285141.

Erik Haugom is holds a PhD in Managerial Economics, Finance and Operations from the Norwegian University of Science and Technology. Today he is employed as a Professor at Inland Norway University of Applied Sciences. His special fields of interest include energy price and volatility modeling and forecasting, risk analysis, risk management, and econometric modeling and forecasting in general.

Iveta Malasevska is a researcher at Eastern Norway Research Institute. She holds a PhD in innovation in services - in the private and public sector at Inland Norway University of Applied Sciences. Malasevska's main research interests cover business forecasting, pricing analytics, revenue optimization, innovative pricing methods, variable pricing, value-based pricing, and tourism in general.

PUBLIC INTEREST STATEMENT

We examine how alpine skiers evaluate various ski resort- and weather-related characteristics. The analysis is based on survey data from 400 existing skiers at a large ski resort in the Inland region of Norway. The weather of the skiing day and the price of a lift ticket are the most important attributes to alpine skiers. Ski lift queue time and the fraction of slopes open are also considered important when visiting the ski resort. This insight can be used directly by ski resort managers when forming new pricing strategies.

© 2019 The Author(s). This open access article is distributed under a Creative Commons Attribution (CC-BY) 4.0 license.

1. Introduction

The alpine skiing industry is a very competitive and vulnerable industry that is facing the challenge of generating long-term growth (Vanat, 2018). First, it is affected by climate change, such as potential lack of snow, insufficient snow depth, and earlier snow melting (Campos Rodrigues, Freire-González, González Puig, & Puig-Ventosa, 2018; Elsasser & Messerli, 2001; Scott, de Freitas, & Matzarakis, 2009). Second, demand for alpine skiing is very variable and dependent on the leisure time, weather conditions, and costs (see e.g., Malasevska, Haugom, & Lien, 2017a). Third, the industry struggles to attract the younger generation that has different consumption patterns, and that is in great demand for all kinds of competing recreation activities (Vanat, 2018). In order to deal with the previously mentioned challenges, and balance the potential short- and long-term effects of different strategies, a deeper understanding of the features important to potential and existing customers when visiting a ski resort should be revealed.

A desire for alpine skiing is interlinked with a ski resort's attributes. The ski resort chosen for a visit has to meet the skier's expectations and provide satisfactory experience in order to be successful. Accordingly, the combination of different attributes results in a ski resort's specific attractiveness among skiers (Gössling, Scott, Hall, Ceron, & Dubois, 2012). However, the overall quality of an alpine skiing experience at a particular ski resort is highly variable and dependent on a variety of attributes that the ski resort cannot directly affect, such as weather conditions or consumers' skiing ability (see Figure 1).

We can assume that skiers choose to visit a ski resort when they can maximize utilities and minimize opportunity costs (Morey, 1984). However, alpine skiing is a special service category (Tsiotsou & Wirtz, 2012). Every time when visiting a ski resort consumers actually make a very difficult decision as the overall service (skiing) experience on a particular day is almost unpredictable. When visiting a chosen ski resort for the first time, a skier relies on outside information, such as advertisements, recommendations from friends, physical characteristics of a ski resort, because he or she cannot rely on any previous personal knowledge. When a skier decides to revisit a particular ski resort, the perception of the attributes of the ski resort becomes more complex and are partially derived from personal experiences (Mangleburg et al., 1998; Perdue, 2000; Sujan, 1985) that are made in three stages; a pre-visitation, visitation and post-visitation stage (Fridgen, 1984; Lovelock & Wirtz, 2011). An unpleasant skiing experience could damage the attractiveness of the destination in the eyes of the consumers and reduce their desire to revisit the ski resort. Accordingly, skier's perceptions of the attractiveness of the ski resort play a significant role in the fluctuation of demand for alpine skiing. However, a ski resort may be able to influence its attractiveness by minimizing the factors that could have a negative impact on the skiers' perception (see Figure 1). For instance, a ski resort could make changes in the pricing approach to compensate for reduced skiing experience on a particular day.

The purpose of this study is to examine the relative importance of attributes that are not directly controlled by the ski resort but which affect the general utility skiers experience when going alpine skiing. The attributes we examine are weather conditions (cloudiness, precipitation, wind,

Figure 1. The role of perception of different attributes of a ski resort in defining ski resort's attractiveness. (Figure adapted from "Consumer behaviour and demand response of tourists to climate change" (Gössling et al., 2012)).

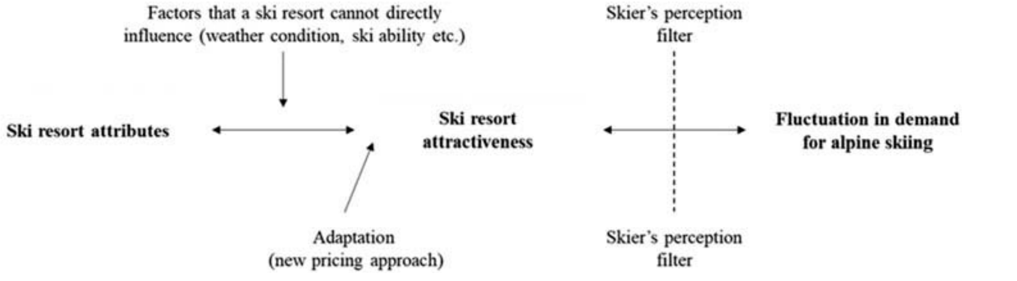

temperature, etc.), the fraction of slopes open, day of the week, and whether it is vacation or a regular week. The analysis is based on survey data from 400 existing skiers at a large ski resort in the Inland region of Norway. To this end, we use conjoint analysis, which allows us to study alpine skiers' preferences and utility functions. The calculations of the relative importance of the various factors when skiers visit a resort could help managers form new and innovative pricing and marketing strategies that, in turn, would increase the utility skiers experience on a particular day and enhance the attractiveness of a ski resort.

Additionally, this study contributes with interesting and more detailed information about skiers' perceptions and provides definitions of the most preferred and least preferred scenario for an alpine skiing day.

The rest of the paper is organized as follows. The next section provides an overview of the literature. Section 3 describes the methodology, the questionnaire design, sample characteristics, and the results of the descriptive statistics. Section 4 presents the empirical results of the conjoint analyses. The final section concludes and provides a discussion about the managerial implications arising from the findings.

2. Literature review

Many previous studies have discussed the relationship between weather- and time-related variables and economic performance of ski resort (see e.g., Falk, 2015; Falk & Vieru, 2017; Gonseth, 2013; Holmgren & McCracken, 2014; Malasevska & Haugom, 2018a; Malasevska et al., 2017a; Morey, 1981; Shih, Nicholls, & Holecek, 2008; Surugiu, Dincă, & Micu, 2010; Töglhofer, Eigner, & Prettenthaler, 2011). The findings of these studies suggest that skiing demand is affected by weather conditions such as air temperature, snow depth, cloudiness and wind chill. Day of the week and holidays also significantly influence skiing demand. Studies of ski destination choice, consumer loyalty, and ski destination attractiveness also find that good snow conditions and snow reliability are among the most important attributes of a ski resort (see e.g., Bausch & Unseld, 2018; Dickson & Faulks, 2007; Godfrey, 1999; Jacobsen, Denstadli, & Rideng, 2008; Kim & Perdue, 2011; Konu, Laukkanen, & Komppula, 2011; Phillips & Brunt, 2013; Richards, 1996; Theodorakis, Tsigilis, & Alexandris, 2009).

In general, ski resorts cannot do anything with the weather conditions or the leisure time of their consumers. However, examining how skiers make trade-offs between various skiing day scenarios enables us to understand what the skiers truly value when visiting a ski resort.

In order to evaluate the importance of the various product- or service attributes, and to simulate consumer decision-making, multi-attribute models (such as conjoint analysis) have been widely used (Tsiotsou & Wirtz, 2012). Still, only a few previous studies have used conjoint analysis within the alpine skiing industry. Carmichael (1996) used conjoint analysis to compute the relative importance of six attributes of a ski resort (variety of runs, snow conditions, value for money, lift lines, staff friendliness and access to home) to improve data collection for market segmentation. Siomkos, Vasiliadis, and Lathiras (2006) applied conjoint analysis to estimate the perceived value across the main service and product attributes offered by 11 ski resorts in Greece. They focused on ski lift and lunch prices, access to the ski resort, parking facilities, service quality and snow quality. They found that the most important attributes for skiers are easy access to the resort and the price of ski lift tickets and lunch, while attributes such as quality, services, facilities and organizational and structural aspects obtained a lower relative importance. Won, Bang, and Shonk (2008) and Won and Hwang (2009) used conjoint analysis to determine factors that influence a skier's or snowboarder's choice of domestic ski destination. Won et al. (2008) collected data from 248 college students (median of 21 years of age). The attributes included in their study were snow quality (below- or above-average), variety of ski trails (15 or 30 trails), travel time (1 or 3 h), expected daily expense, and amenities including restaurants, shopping, and accommodation (limited or plenty). The results showed that snow quality is the most important factor for selecting a ski destination for both skiers and snowboarders, no matter whether they are one-day visitors or

multi-day visitors. In turn, the relative importance of other factors varied largely depending on both recreation specialization (skiing vs. snowboarding) and consumption situation (one-day vs multi-day visit). Won and Hwang (2009) investigated the relative importance of the snow quality (rated on a 5-point scale), average lift waiting time, travel time (1 or 3 h), daily expense (including lift price, lunch, and other fees), and the number of ski slopes (10 or 20 slopes). While similar to the finding of Won et al. (2008), snow quality was emphasised as the most important factor among all visitors. They also found that skiers prioritize snow quality to a greater extent than snowboarders.

None of the previous studies, even those that have used conjoint analysis in the context of alpine skiing industry, has examined *in detail* the relative importance of weather-related and time-related attributes, and the scenarios for the most desirable- and worst skiing day, as we do in this paper.

3. Methods

3.1. Conjoint analysis

A conjoint analysis is an attribute-based survey method that measures the consumers trade-offs among multi-attribute products. This technique helps to determine an individual's value system, which specifies what attributes, exactly, do consumer consider when purchasing within the product- or service category of interest. It is not always easy for respondents to state their value system reliably. Therefore, the great advantage of conjoint analysis is that, rather than asking respondents to value separately individual attributes and attribute levels (i.e., different expressions of the attributes), this technique allows to ask them to make judgement about combinations of attributes (Malhotra & Briks, 2007). In this way, respondents are required to make trade-offs between product attributes that are similar to trade-offs they make in the real marketplace, making it possible to learn what the skiers truly value when deciding to visit a ski resort. Subsequently, conjoint analysis allows us to investigate the preference of skiers for each attribute level.

During the conjoint analysis, a numerical part-worth utility for each attribute level and the importance of each attribute, regardless of its level, are computed. Higher utility values indicate greater preference while small utilities denote the least preferred levels of each attribute. Since the utilities are all expressed in a common unit, they can be added together to give the total utility of any combination of attributes.

There are different procedures available for estimating the basic conjoint model. In the current study, we have obtained metric data, the ratings of various alternative scenarios, and the most appropriate data analysis is therefore OLS regression with dummy variables (Malhotra & Briks, 2007):

$$Y_i = \beta_o + \beta_i X_i + e_i \tag{1}$$

where $Y_i$is a vector of skiers preference rating, X_i is a matrix of dummy variables for the attribute levels. The estimated coefficients of the OLS regression can be related to the part-worths. Given the dummy variable coding, each dummy variable coefficient represents the difference in the average ratings for that level of the attribute, relative to the average rating for the base level.

The mathematical model expressing the fundamental relationship between attributes and utility in conjoint analysis is given by:

$$U(X) = \sum_{i=1}^{m} \sum_{j=1}^{k_i} a_{ij} x_{ij} \tag{2}$$

where $U(X)$ is overall utility of an alternative, a_{ij} is the part-worth contribution or utility associated with the jth level of the ith attribute, k_i is number of levels of attribute i, and m is number of attributes. $x_{ij} = 1$ if the jth level of the ith attribute is present, otherwise x_{ij}= 0.

The importance of an attribute is defined in terms of the range of the part-worth (a_{ij}) across the levels of that attribute:

$$I_i = \{\max(a_{ij}) - min(a_{ij})\}\ for\ each\ i \tag{3}$$

The attribute's importance is normalised to ascertain its importance relative to other attributes:

$$W_i = \frac{I_i}{\sum_{i=1}^{m} I_i} \tag{4}$$

so that

$$\sum_{i=1}^{m} W_i = 1 \tag{5}$$

Hence, the quantity obtained in Equation (4) presents the *relative importance* of an attribute and can be compared directly with other attributes examined in the study.

3.2. Questionnaire design, and data preparation

In a conjoint questionnaire, the respondent evaluates the scenario for a given day at ski resort based on the considered attributes and gives the highest rating to the option that would provide him or her the highest total utility. Therefore, it is important to include all attributes that can influence the total utility of the respondent in order to simulate the real decision-making process. However, including all attributes that could be relevant when skiers consider the attractiveness of visiting the ski resort on a given day would induce a very complex questionnaire. Based on the purpose of our study and the review of previous studies within the alpine skiing industry, the following attributes are included: weather conditions (including temperature and wind), the share of ski slopes open, waiting time at the main lifts, weekday, vacation, and price. Table 1 shows all the attributes and attribute levels included in this survey.

Various versions of the questionnaire were pre-tested before conducting the final survey. To this end, we used a small sample (approximately 15 respondents in total) consisting of students and colleagues at the university college to evaluate several versions of the questionnaire. In the pre-test, we soon realized that there would be a trade-off between complexity and the number of potentially relevant attributes to include. To overcome the complexity problem while retaining the opportunity of a rich set of attributes to include, we created questionnaires that differed slightly on some of the characteristics we wanted to measure. This was done by changing some of the information before the respondent should reveal his/her ratings of how likely it was that he/she would go skiing in the various scenarios. The information that differed from the various versions of the questionnaire was the weather (using different weather symbols), fractions of slopes open, and the time-period for which the skiing activity was going to take place (regular week or vacation). The rest of the attributes, and their levels, were all reflected in the two tables with nine profiles each. Figure 2 illustrates how question 8 looks like for two different versions of the questionnaire.

The difference between these two versions is the weather of the skiing day. In the upper panel, the weather symbols indicate that it is a day with snowfall, 0°C, and no wind. In the lower panel, the symbols illustrate a day with nice sunny weather, −5°C, and no wind. This way of collecting the data allows us to create a data set where the relative importance of many attributes can be analyzed without overwhelming all respondents with questions about all the attribute levels. In total, we created 10 versions of the same questionnaire, where weather-, time-period, or slopes-characteristics (question 8 and 9) differs slightly. Table 2 presents the features of the key questions (Q8 and Q9) for all 10 questionnaires.

3.3. Sample and descriptive statistics

The sample consists of active skiers at one major ski resorts in the inland region of Norway.[1] Table 3 provides detailed information about the main features of the ski resort. The data collection was

Table 1. Skiing day attributes included in the survey

Attribute	Attribute level
Weather conditions	Sunny
	Snow
	Cloudy
	Foggy
	Rain
Temperature	−20 C°
	−15 C°
	−10 C°
	−5 C°
	0 C°
	5 C°
Wind	No wind
	Gentle breeze
	Fresh breeze
Share of slopes open	50%
	75%
	100%
Waiting time in the main lift	1 min
	5 min
	10 min
Weekday	Midweek
	Weekend
Vacation	Regular week
	Holiday season
Price	NOK 250
	NOK 350
	NOK 450
	NOK 550
	NOK 650

performed by four bachelor students during March and April 2018. The students collected the data at the ski resort at various weekdays (including weekends) and during the Easter holiday. A total of 400 forms were either partly or fully completed. The total number of obtained ratings (Q8 and Q9) amounts to 6490. As each respondent was asked to perform 18 ratings, the average number of ratings performed by each respondent is 16.23 or 90.14%. A total of 350 out of the 400 respondents had completed all 18 ratings, which corresponds to 87.5%. For conjoint analysis, a sample size of 150 is required to ensure reliability (Orme, 2010). Thus, the sample size of the current study meets the minimum requirement to ensure reliable results of the conjoint analysis. Descriptive statistics of the sample are given in Table 4. The reported numbers correspond well with previous studies in the same area (see, e.g., Malasevska & Haugom, 2018b). Approximately 60% of the respondents are from Norway and 6 out of 10 skiers/snowboarders report a household income of more than NOK 600 000. The most popular equipment when visiting the ski resort is alpine skies (almost 8 out of 10 use this) followed by snowboard (15%).

Table 5 shows the average rating across the various profiles (from 1 to 9) on question 8 and 9, respectively. In general, the skiing day characteristics are poorer on question 9 compared to those presented in question 8.[2] The results show that the highest average ratings are obtained for low

cogent • social sciences

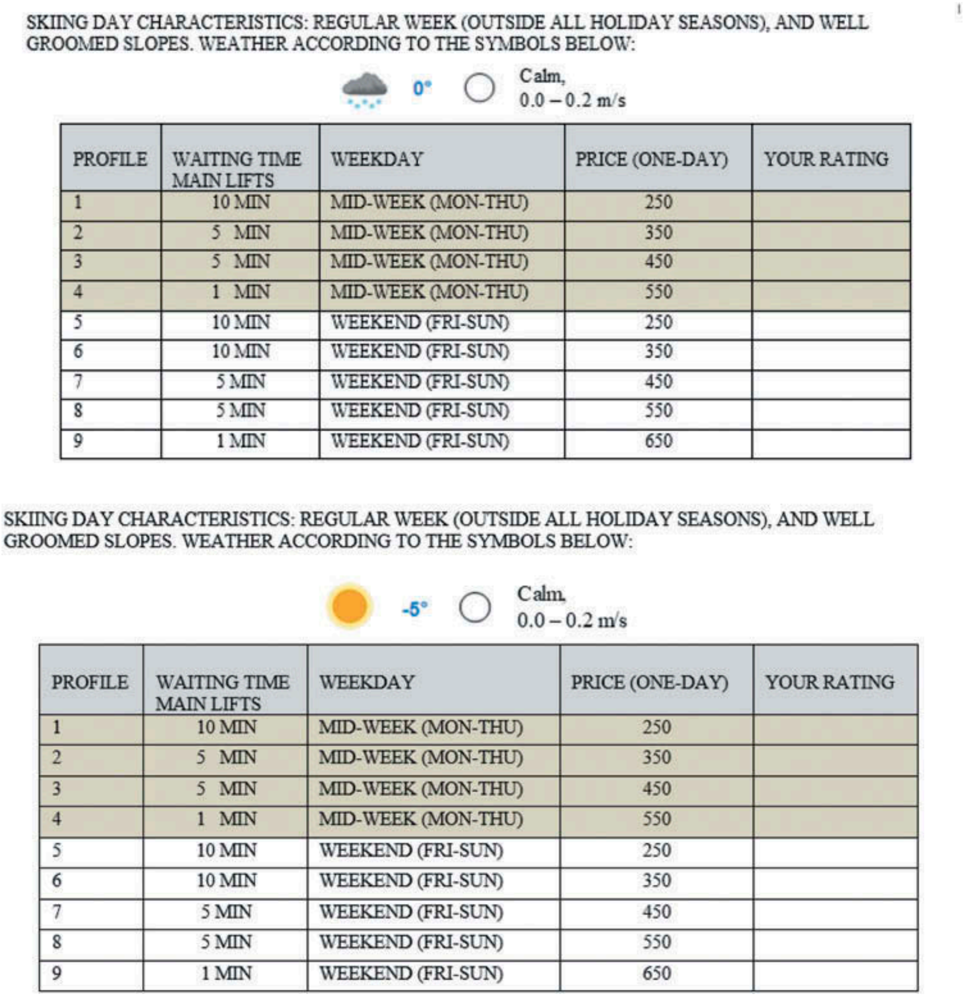

SKIING DAY CHARACTERISTICS: REGULAR WEEK (OUTSIDE ALL HOLIDAY SEASONS), AND WELL GROOMED SLOPES. WEATHER ACCORDING TO THE SYMBOLS BELOW:

0° Calm, 0.0 – 0.2 m/s

PROFILE	WAITING TIME MAIN LIFTS	WEEKDAY	PRICE (ONE-DAY)	YOUR RATING
1	10 MIN	MID-WEEK (MON-THU)	250	
2	5 MIN	MID-WEEK (MON-THU)	350	
3	5 MIN	MID-WEEK (MON-THU)	450	
4	1 MIN	MID-WEEK (MON-THU)	550	
5	10 MIN	WEEKEND (FRI-SUN)	250	
6	10 MIN	WEEKEND (FRI-SUN)	350	
7	5 MIN	WEEKEND (FRI-SUN)	450	
8	5 MIN	WEEKEND (FRI-SUN)	550	
9	1 MIN	WEEKEND (FRI-SUN)	650	

SKIING DAY CHARACTERISTICS: REGULAR WEEK (OUTSIDE ALL HOLIDAY SEASONS), AND WELL GROOMED SLOPES. WEATHER ACCORDING TO THE SYMBOLS BELOW:

-5° Calm, 0.0 – 0.2 m/s

PROFILE	WAITING TIME MAIN LIFTS	WEEKDAY	PRICE (ONE-DAY)	YOUR RATING
1	10 MIN	MID-WEEK (MON-THU)	250	
2	5 MIN	MID-WEEK (MON-THU)	350	
3	5 MIN	MID-WEEK (MON-THU)	450	
4	1 MIN	MID-WEEK (MON-THU)	550	
5	10 MIN	WEEKEND (FRI-SUN)	250	
6	10 MIN	WEEKEND (FRI-SUN)	350	
7	5 MIN	WEEKEND (FRI-SUN)	450	
8	5 MIN	WEEKEND (FRI-SUN)	550	
9	1 MIN	WEEKEND (FRI-SUN)	650	

Figure 2. Illustration of how question 8 differs for two versions of the questionnaire.

Table 2. The 10 versions of the questionnaire used in the survey

Code	Weather	Temp	Wind	Period	Slopes open	Change from Q8 to Q9
SLOPES-1	Sun	−5	No wind	Regular week	100 %	Slopes open: 75 %
SLOPES-2	Sun	−5	No wind	Regular week	100 %	Slopes open: 50 %
RAIN-1	Rain	5	No wind	Regular week	100 %	Wind: Gentle breeze
SNOW-1	Snow	0	No wind	Regular week	100 %	Wind: Gentle breeze
SNOW-2	Snow	−15	No wind	Regular week	100 %	Wind: Gentle breeze
TEMP-1	Sun	−5	No wind	Regular week	100 %	Temperature: −15
TEMP-2	Sun	5	No wind	Regular week	100 %	Temperature: −20
TP-1	Sun	−5	No wind	Regular week	100 %	Period: Vacation
WIND-1	Cloudy	−10	No wind	Regular week	100 %	Wind: Fresh breeze
WIND-2	Fog	0	No wind	Regular week	100 %	Wind: Fresh breeze

prices during the weekend, and mostly for low to medium waiting times in the main lifts. However, these results are aggregates across many different weather scenarios and to fully evaluate the relative importance of all characteristics, and the utilities attached to the levels of these, we must apply the conjoint method. The results of these analyses are given in the next section.

Table 3. The characteristics of the ski resort

Characteristic	Value
Total ski lift capacity (persons per hour)	21,060
Total length of ski slopes (km)	44
Number of ski slopes	32
Number of ski lifts	18
Base altitude of ski resort (m)	195
Vertical drop (m)	835

4. Results

4.1. Relative importance of attributes

Figure 3 shows the calculated relative importance of the various attributes examined in this study. The relative importance is calculated as described in section 2. The results clearly show that the *price* is the single most important attribute when visiting the resort. In our study, the relative importance of price is calculated to 35%. Our results are in line with findings of Siomkos et al. (2006) who have investigated seven different attributes and found that the lift ticket price is one of the most valued attributes with a relative importance 19.44%. Won et al. (2008) and Won and Hwang (2009) did not include price directly when calculating the relative importance but instead used an attribute called *daily cost*, which included ski lift ticket, food, and other fees. In addition, Won et al. (2008) divided respondents into two main subgroups: one-day visitors and multi-day visitors. Their results suggested that the relative importance of daily cost attribute among five attributes for one-day visitors and multi-day visitors was 19.6% and 13.8%, respectively. For comparison, the relative importance of the *daily cost* attribute in their study was calculated to 16.2%.

Weather-related attributes follow as the next most important to skiers when visiting the ski resort. The sum of the relative importance for the three weather-related attributes *weather (cloudiness/precipitation), temperature*, and *wind* amounts to 33%. Among the different weather variables, temperature is the most prominent one having a relative importance of 14%. Wind and cloudiness/precipitation are weighted almost identically among the skiers at this resort. Won and Hwang (2009) did not examine the importance of weather, but our findings are in line with those of Malasevska, Haugom, and Lien (2017b) who find substantially lower optimal prices during periods of bad weather conditions. We will return to this when we examine the part-worth functions next.

The fraction of the number of slopes open and waiting time in the main lifts (queue) are also relatively important to skiers when visiting the resort. In our study, we calculated the relative importance of both these two attributes to 12%. For comparison, Won and Hwang (2009), found that waiting time in the lifts was the second most important attribute (relative importance of 18.2%) for ski resorts in Korea.

The least important attributes in this study are *weekday* (6%) and *vacation* (2%). Both these attributes only assume two levels (midweek/weekend and vacation/regular week). It is therefore not surprising that the difference between the minimum and maximum level of the part-worth for these attributes is somewhat narrower when compared to the other attributes that have three or more levels. It is interesting to note, however, that weekday is perceived substantially more important than whether it is vacation or a regular week.

4.2. Part-worth utilities

The part-worth functions for all the examined attributes are graphed in Figures 3 and 4. In Figure 3, we focus on queue time in the main lift (upper left panel), price (upper right panel), weekday (lower left panel) and whether it is vacation or regular week (lower right panel). As expected, the utility is highest

Table 4. Descriptive statistics

**Variable**	
N	400
Gender (%)	
Male	57.00
Female	43.00
Total	100.00
Nationality (%)	
Norwegians	61.25
Other	38.75
Total	100.00
Average age (years)	34.74 (median = 35.00, SD = 12.74)
Net income (%)	
Below NOK 100 000	5.75
NOK 100 000—NOK 300 000	8.75
NOK 300 001—NOK 600 000	15.50
NOK 600 001—NOK 900 000	19.00
NOK 900 001—NOK 1 200 000	12.50
More than NOK 1 200 000	13.75
Prefer not to answer	24.75
Total	100.00
Family status (%)	
Single	25.75
Single with children	1.50
Couple	28.75
Couple with children	39.75
Other	1.75
Prefer not to answer	2.50
Total	100.00
Current occupation (%)	
Working full time	66.50
Working part time	9.75
Unemployed	0.00
Student	18.25
Other	3.50
Prefer not to answer	2.00
Total	100.00
Main recreational activity when visiting a ski resort	
Alpine skiing	78.50
Snowboarding	
Other	3.75
Prefer not to answer	2.50
Total	100.00
Skiing/snowboarding interest (measured on a Likert scale from 1 to 7)	5.19 (median = 5.00, SD = 1.42)

Table 5. Mean rating of the 9 different profiles for question 8 and 9 across all other skiing day characteristics

Profile	Waiting time main lifts	Weekday	Price (one-day)	Rating Q8	Rating Q9
1	10 MIN	MID-WEEK (MON-THU)	250	55.6	45.1
2	5 MIN	MID-WEEK (MON-THU)	350	60.4	47.8
3	5 MIN	MID-WEEK (MON-THU)	450	47.0	37.2
4	1 MIN	MID-WEEK (MON-THU)	550	38.9	32.4
5	10 MIN	WEEKEND (FRI-SUN)	250	64.2	50.3
6	10 MIN	WEEKEND (FRI-SUN)	350	58.3	45.8
7	5 MIN	WEEKEND (FRI-SUN)	450	56.4	44.0
8	5 MIN	WEEKEND (FRI-SUN)	550	41.6	32.5
9	1 MIN	WEEKEND (FRI-SUN)	650	33.7	26.2

Figure 3. The calculated relative importance of the various attributes when visiting a ski resort.

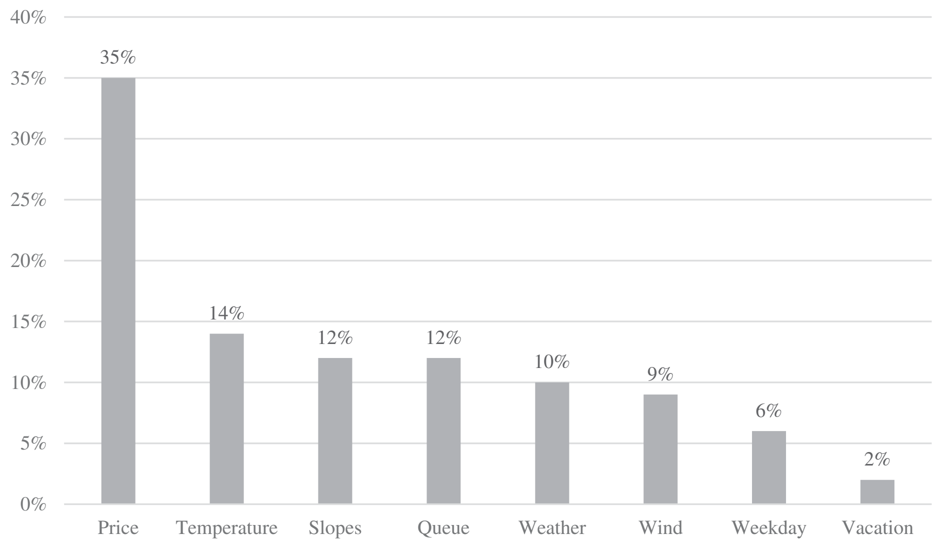

for short waiting times in the main lifts. One-minute waiting time is clearly most preferred, also when controlling for the other attributes in this study. The utility estimate for waiting time also indicates a steeper decline in utility when moving from 5 to 10 min, compared to moving from 1 to 5. This provides some initial insight on the potential negative consequences of pricing alpine skiing tickets "too low". As lower prices induce more traffic in the ski resorts, there is a direct link also between lower prices and the reduced utility skiers experience from longer waiting lines.

The part-worth function for price exhibit a distinct downward-sloping pattern. The higher the price, the lower is the average utility. This function also has a distinct "kink" at NOK 350, where the slope of

cogent • social sciences

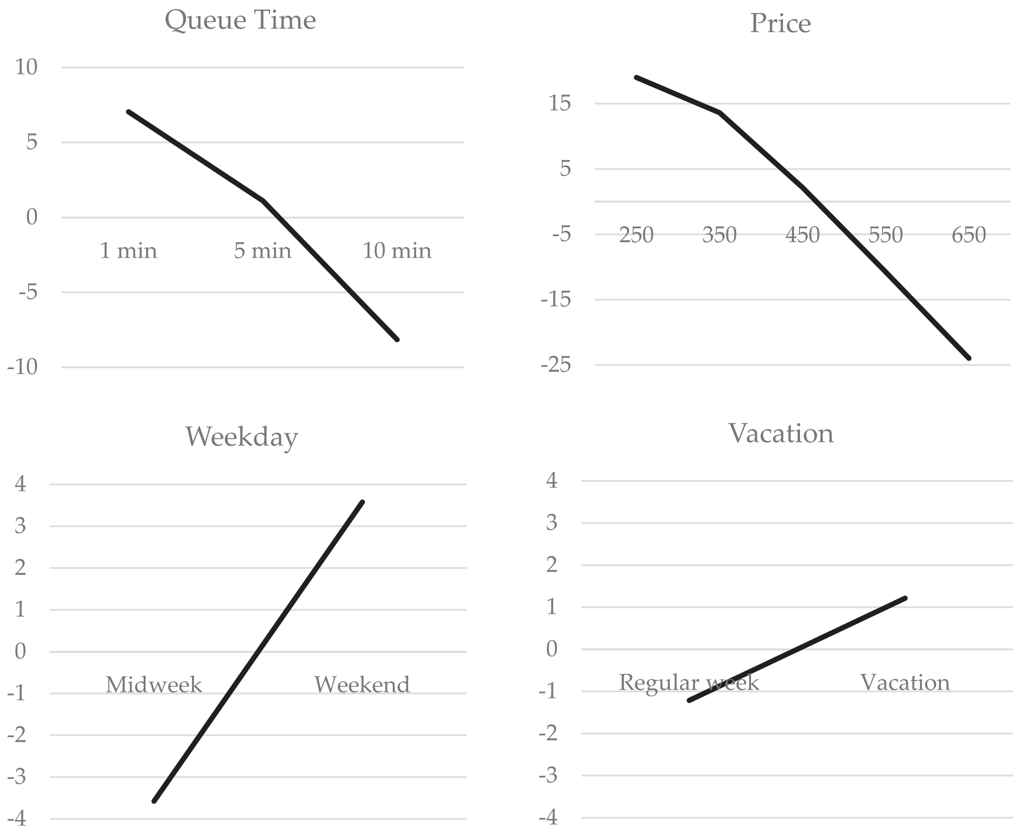

Figure 4. The calculated part-worth functions for four of the variables.

the part-worth curve is steeper for prices higher than this compared to prices lower than this. Hence, the skiers of this resort clearly become more price-sensitive for prices higher than NOK 350.

In the lower panel of Figure 4, we illustrate the part-worth functions related to what time period it is. Clearly, skiers prefer skiing during the weekend compared to the midweek. The difference in the calculated utility from these two intra-weekly periods is quite big, at least compared to the difference across regular weeks and vacations (right panel). If combining the utility of the various prices with the weekday utility, a price of 250 for a midweek day would yield approximately the same utility as a price of 360 in the weekend, on average, and everything else equal. This result strongly supports Malasevska and Haugom (2018b) who find a substantial lower optimal (short-term) price for midweek days (Monday—Thursday) compared to the weekend.

The skiers of this resort are not very sensitive to whether it is vacation or a regular week, though. This could potentially be explained by the number of local skiers compared to tourists. The resort of interest has a high fraction of local skiers and these have the freedom to visit the resort during the whole season. Nevertheless, our results show that there is some difference in preferences across time-period (vacation vs regular week). And; if there is excess capacity in weeks outside the main vacation periods (Christmas holiday, winter holiday, eastern holiday), ski resorts could use price to “balance” the utility skiers experience by lowering prices during these “off-season” periods.

Figure 5 focuses on weather- and weather-related variables. The upper left panel shows the part-worth function for the weather as measured by cloudiness and precipitation. The highest utility is clearly observed for clear/stable sky. Not surprisingly, skiers then prefer snow to foggy weather and rain. Again, there is a very distinctive kink when moving from snow to fog/rain. Hence, the latter weather is perceived substantially worse for skiing activities in general.

Figure 5. The calculated part-worth functions for four of the variables.

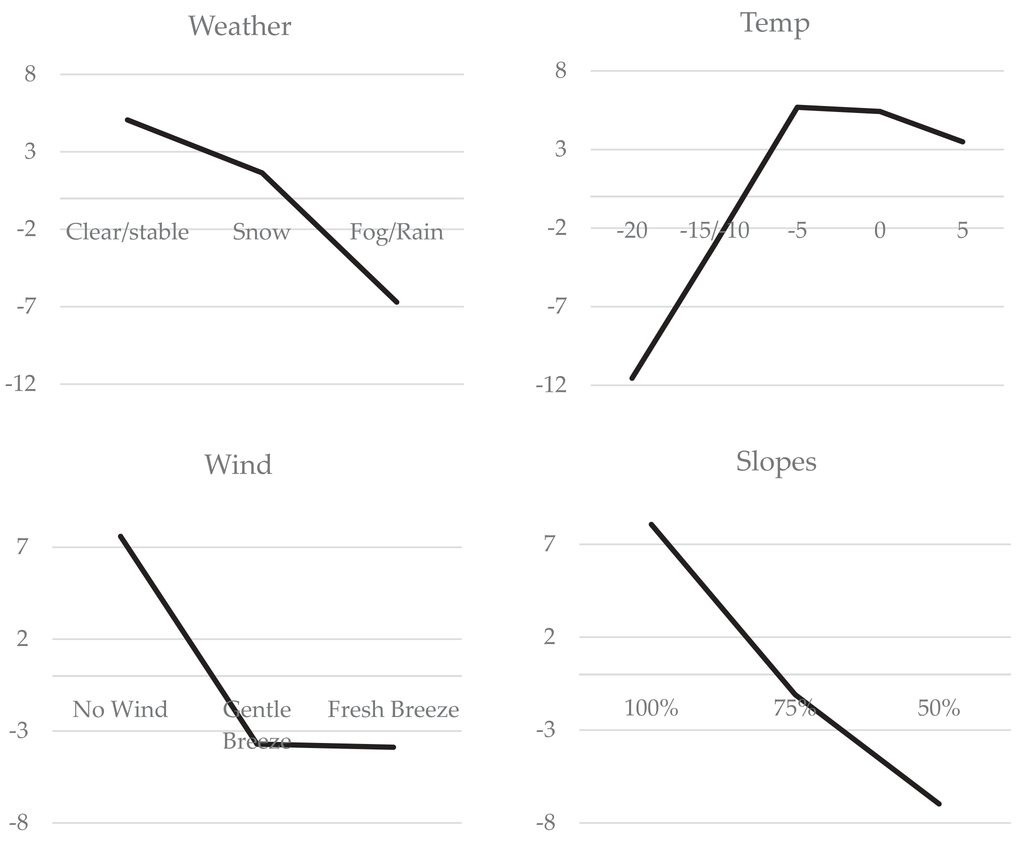

The upper right panel of Figure 4 shows the temperature-preference for skiers at this resort. The utility increases rapidly with increased temperatures from −20 C° and up to −5 C°. From that point, the overall utility starts to decline slowly from −5 C° to 0 C°, and somewhat faster from 0 C° to 5 C°. This figure indirectly states that the "optimal" (utility maximizing) skiing temperature is about −5 C°. This finding seems reasonable and corresponds well with the results of Malasevska et al. (2017a) who find an optimal *wind child* temperature of approximately −9.5 C°.[3]

The lower left panel shows the part-worth function for the wind variable. Clearly, skiers are sensitive to the wind conditions when visiting the resort. There is a substantial higher calculated utility during *no wind* scenarios compared to scenarios with either *gentle breeze* or *fresh breeze*. The utility drops rapidly when moving from *no wind* to *gentle breeze*, while the part-worth function flattens out when moving from *gentle breeze* to *fresh breeze*. This shape of the function makes sense from a practical point of view, as skiing conditions in practice move from very good to bad (no wind to gentle breeze) in the first case, and from bad to worse (gentle breeze to fresh breeze) in the second case. Hence, this finding suggests that *gentle breeze* is enough to induce pretty bad skiing conditions and that increasing the wind beyond this will have a little impact on the overall utility (on the margin). The finding of substantial lower utility for windy conditions also corresponds well with the results of Malasevska et al. (2017b) who find substantial lower optimal prices for such conditions.

The lower right panel presents the part-worth function for the various levels of the fraction of slopes that are open. Not surprisingly, the highest utility is obtained when all slopes are open. The utility associated with this variable has a steeper decline when moving from 100% to 75% compared to moving from 75% to 50%. Hence, skiers are less sensitive about reducing the capacity when some of the slopes are already closed. In general, however, the overall utility skiers

Table 6. The most preferred and the least preferred weather-related conditions for a skiing day

	The most preferred scenario	The least preferred scenario
Weather conditions	Sunny or stable conditions with no precipitation	Fog/bad visibility
Air temperature	−5°C	−20°C
Wind	No wind	Fresh breeze
Slopes open (snow reliability)	All slopes are open	50% of slopes are closed

experience when visiting the resort is highly affected by how much of the total capacity of the ski resort that is available for them to enjoy.

Based on the part-worth functions, we can determine the most preferred and the least preferred weather-related conditions for a skiing day. These scenarios are presented in Table 6. These findings are important when ski resort managers are forming pricing schemes based on the weather-related attributes.

5. Conclusion and implications

The main purpose of this study has been to examine how skiers and snowboarders evaluate the importance of various ski resort- and weather-related attributes when visiting a ski resort. Our results suggest that the price level, weather-related attributes, the fraction of slopes open, and waiting time in the main lifts strongly influence the utility skiers and snowboarders experience when visiting the ski resort. We also find that the weekday (midweek vs weekend) and whether it is vacation or a regular week have some influence on the utility.

Apart from the price level itself, the weather-related attributes are considered the most important when evaluating the attractiveness of a skiing day in this study. All the weather-related attributes combined has a relative importance of 35%. Temperature is considered most important (14%) followed by cloudiness/precipitation (10%) and wind (9%). Though the importance of weather has been documented in previous research on demand for alpine skiing (e.g., Falk, 2013; Hamilton, Brown, & Keim, 2007; Holmgren & McCracken, 2014; Malasevska et al., 2017a; Shih et al., 2008; Töglhofer et al., 2011), it has not been documented how it affects the revealed utility of skiers. Hence, our study contributes to the existing knowledge by providing a deeper understanding of how the weather conditions, including the wind conditions, are evaluated relative to other attributes.

Waiting time in the main-lifts and the fraction of slopes open are also important to skiers and snowboarders (both have a relative importance of 12%). Our results suggest that the utility skiers experience when ski lift waiting times are above 5 min, and when only 75% or less of the slopes are open, drops substantially. The time period when the skiing takes place also has some effect on the overall utility. In general, weekend skiing is preferred over midweek skiing, while the utility skiers experience is slightly higher during vacations compared to regular weeks.

All these findings provide key insights that can directly help managers evaluate how the “skiing day utility” of their customers are formed. In turn, this can be used as important decision support when forming new pricing strategies and in operational planning more generally. For example, if all skiing days are priced the same, visitors would tend to crowd into the most desirable (utility maximizing) days, while a lot of capacity would be left unused on less attractive skiing days. The simplest way to balance the utility skiers experience is to adjust the price such that it better matches the perceived quality of the alpine skiing on a particular day. The results of the current study can be used as input when doing this balancing in an optimization framework.

Previous research has shown that ski resorts have the potential to increase their revenues by adopting a more dynamic approach to pricing (Malasevska & Haugom, 2018b), including offering weather-related discounts (Malasevska et al., 2017b). Some ski resorts in Switzerland (e.g., Pizol and Valais) have already begun to implement such variable pricing by offering a reduction in one-day ski lift ticket prices if the weather conditions are bad.

The price of a ski lift ticket is the aggregate of all attributes of the offered skiing experience and services (Alessandrini, 2013). Therefore, a more dynamic approach to pricing may positively influence the market positions of ski resorts, while simultaneously increasing skiers' satisfaction, as they will feel that the skiing experience meets their expectation and is worth the money paid.

Dynamic pricing that is directly based on the historical, current, or forecasted weather, is possibly most suitable for one-day ski passes. However, ski resorts also have visitors that purchase multi-day ski passes. To make dynamic pricing fair to all visitors, ski resorts should consider some "bad weather" benefits for multi-day visitors as well, because it will increase the chance that the visitor will be more satisfied with the overall skiing experience, and in turn will decide to revisit the ski resort. Bad weather benefits could be provided in cooperation with other local businesses, such as accommodation, restaurants, non-skiing activities, etc., as a decrease in alpine skiing demand is not an issue only for the ski resort itself but also for nearby local businesses.

Future research should explore additional sets of attributes. In addition, it could be interesting to examine whether the relative importance of different attributes varies between consumer sub-groups, which would make some new niche marketing- and pricing strategies possible.

Funding
This work is part of the project "Innovative Pricing Approaches in the Alpine Skiing Industry" and supported by the Regionale Forskningsfond Innlandet [285141].

Author details
Erik Haugom[1]
E-mail: erik.haugom@inn.no
Iveta Malasevska[2]
E-mail: im@ostforsk.no
[1] Inland School of Business and Social Sciences, Inland Norway University of Applied Sciences, Elverum, 2418, Norway.
[2] Inland School of Business and Social Sciences, Eastern Norway Research Institute, Inland Norway University of Applied Sciences, Elverum, 2418, Norway.

Citation information
Cite this article as: The relative importance of ski resort- and weather-related characteristics when going alpine skiing, Erik Haugom & Iveta Malasevska, *Cogent Social Sciences* (2019), 5: 1681246.

Notes

1. Due to the competitiveness in the Norwegian alpine skiing industry, the ski resort has requested to stay anonymous.
2. The exception is the questionnaire examining the effect of vacation vs. regular week. See Table 1 for details.
3. As the name suggests, the wind chill measure also considers the wind. The wind chill can be calculated using the following formula: $W = 13.12 + 0.6215 \times T - 11.37 \times V^{0.16} + 0.3965 \times T \times V^{0.16}$.

References

Alessandrini, S. (2013). Quality of ski resorts and competition between the Emilian Apennines and Altipiani Trentini. An estimate of the hedonic price. *Review of Economic Analysis*, *5*(1), 42–69.

Bausch, T., & Unseld, C. (2018). Winter tourism in Germany is much more than skiing! Consumer motives and implications to Alpine destination marketing. *Journal of Vacation Marketing*, *24*(3), 203–217. doi:10.1177/1356766717691806

Campos Rodrigues, L., Freire-González, J., González Puig, A., & Puig-Ventosa, I. J. C. (2018). Climate change Adaptation of Alpine Ski Tourism in Spain. *Climate*, *6*(2), 29. doi:10.3390/cli6020029

Carmichael, B. A. (1996). Conjoint analysis of downhill skiers used to improve data collection for market segmentation. *Journal of Travel & Tourism Marketing*, *5*(3), 187–206. doi:10.1300/J073v05n03_02

Dickson, T. J., & Faulks, P. (2007). Exploring overseas snowsport participation by Australian skiers and snowboarders. *Tourism Review*, *62*(3/4), 7–14. doi:10.1108/16605370780000315

Elsasser, H., & Messerli, P. (2001). The vulnerability of the snow industry in the Swiss Alps. *Mountain Research and Development*, *21*(4), 335–339. doi:10.1659/0276-4741(2001)021[0335:TVOTSI]2.0.CO;2

Falk, M. (2013). Impact of long-term weather on domestic and foreign winter tourism demand. *International Journal of Tourism Research*, *15*(1), 1–17. doi:10.1002/jtr.v15.1

Falk, M. (2015). The demand for winter sports: Empirical evidence for the largest French ski-lift operator. *Tourism Economics*, *21*(3), 561–580. doi:10.5367/te.2013.0366

Falk, M., & Vieru, M. (2017). Demand for downhill skiing in subarctic climates. *Scandinavian Journal of Hospitality and Tourism*, *17*(4), 388–405. doi:10.1080/15022250.2016.1238780

Fridgen, J. D. (1984). Environmental psychology and tourism. *Annals of Tourism Research*, *11*(1), 19–39. doi:10.1016/0160-7383(84)90094-X

Godfrey, K. B. (1999). Attributes of destination choice: British skiing in Canada. *Journal of Vacation Marketing*, *5*(1), 18–30. doi:10.1177/135676679900500103

Gonseth, C. (2013). Impact of snow variability on the Swiss winter tourism sector: Implications in an era of climate change. *Climatic Change*, *119*(2), 307–320. doi:10.1007/s10584-013-0718-3

Gössling, S., Scott, D., Hall, C. M., Ceron, J.-P., & Dubois, G. (2012). Consumer behaviour and demand response of tourists to climate change. *Annals of Tourism Research*, *39*(1), 36–58. doi:10.1016/j.annals.2011.11.002

Hamilton, L. C., Brown, C., & Keim, B. D. (2007). Ski areas, weather and climate: Time series models for New England case studies. *International Journal of Climatology*, *27*(15), 2113–2124. doi:10.1002/(ISSN)1097-0088

Holmgren, M. A., & McCracken, V. A. (2014). What affects demand for "the greatest snow on earth?". *Journal of Hospitality Marketing & Management*, *23*(1), 1–20. doi:10.1080/19368623.2012.746212

Jacobsen, J. K. S., Denstadli, J. M., & Rideng, A. (2008). Skiers' sense of snow: Tourist skills and winter holiday attribute preferences. *Tourism Analysis*, *13*(5/6), 605–614. doi:10.3727/108354208788160478

Kim, D., & Perdue, R. R. (2011). The influence of image on destination attractiveness. *Journal of Travel & Tourism Marketing*, *28*(3), 225–239. doi:10.1080/10548408.2011.562850

Konu, H., Laukkanen, T., & Komppula, R. (2011). Using ski destination choice criteria to segment Finnish ski resort customers. *Tourism Management*, *32*(5), 1096–1105. doi:10.1016/j.tourman.2010.09.010

Lovelock, C., & Wirtz, J. (2011). *Services marketing: People, technology, strategy* (7th ed.). Upper Saddle River, New Jersey: Prensice Hall.

Malasevska, I., & Haugom, E. (2018a). Alpine skiing demand patterns. *Scandinavian Journal of Hospitality and Tourism*, 1–14. doi:10.1080/15022250.2018.1539924

Malasevska, I., & Haugom, E. (2018b). Optimal prices for alpine ski passes. *Tourism Management*, *64*, 291–302. doi:10.1016/j.tourman.2017.09.006

Malasevska, I., Haugom, E., & Lien, G. (2017a). Modelling and forecasting alpine skier visits. *Tourism Economics*, *23*(3), 669–679. doi:10.5367/te.2015.0524

Malasevska, I., Haugom, E., & Lien, G. (2017b). Optimal weather discounts for alpine ski passes. *Journal of Outdoor Recreation and Tourism*, *20*, 19–30. doi:10.1016/j.jort.2017.09.002

Malhotra, N. K., & Briks, D. F. (ed.). (2007). *Marketing research: An applied approach* (3rd ed.). Essex, UK: Pearson Education Limited.

Mangleburg, T. F., Sirgy, M. J., Grewal, D., Axsom, D., Hatzios, M., Claiborne, C., & Bogle, T. (1998). The moderating effect of prior experience in consumers' use of user-image based versus utilitarian cues in brand attitude. *Journal of Business and Psychology*, *13*(1), 101–113. doi:10.1023/A:1022927201433

Morey, E. R. (1981). The demand for site-specific recreational activities: A characteristics approach. *Journal of Environmental Economics and Management*, *8*(4), 345–371. doi:10.1016/0095-0696(81)90046-2

Morey, E. R. (1984). The choice of ski areas: Estimation of a generalized CES preference ordering with characteristics. *The Review of Economics and Statistics*, *66*, 584–590. doi:10.2307/1935982

Orme, B. K. (2010). *Getting started with conjoint analysis: Strategies for product design and pricing research* (2nd ed.). Madison, Wis: Research Publishers, LLC.

Perdue, R. R. (2000). Destination images and consumer confidence in destination attribute ratings. *Tourism Analysis*, *5*(2–3), 77–81.

Phillips, J., & Brunt, P. (2013). Tourist differentiation: Developing a typology for the winter sports market. *Turizam: znanstveno-strucni Casopis*, *61*(3), 219–243.

Richards, G. (1996). Skilled consumption and UK ski holidays. *Tourism Management*, *17*(1), 25–34. doi:10.1016/0261-5177(96)00097-0

Scott, D., de Freitas, C., & Matzarakis, A. (2009). Adaptation in the tourism and recreation sector. In K. L. Ebi, I. Burton, & G. R. McGregor (Eds.), *Biometeorology for adaptation to climate variability and change* (pp. 171–194). Dordrecht: Springer Netherlands.

Shih, C., Nicholls, S., & Holecek, D. F. (2008). Impact of weather on downhill ski lift ticket sales. *Journal of Travel Research*, *47*(3), 359–372. doi:10.1177/0047287508321207

Siomkos, G., Vasiliadis, C., & Lathiras, P. (2006). Measuring customer preferences in the winter sports market: The case of Greece. *Journal of Targeting, Measurement Analysis for Marketing*, *14*(2), 129–140. doi:10.1057/palgrave.jt.5740175

Sujan, M. J. (1985). Consumer knowledge: Effects on evaluation strategies mediating consumer judgments. *Journal of Consumer Research*, *12*(1), 31–46. doi:10.1086/jcr.1985.12.issue-1

Surugiu, C., Dincă, A.-I., & Micu, D. (2010). Tourism destinations vulnerable to climate changes: An econometric approach on Predeal resort. *Economic Science Series*, *62*(1), 111–120.

Theodorakis, N. D., Tsigilis, N., & Alexandris, K. (2009). The mediating role of place attachment on the relationship between service quality and loyalty in the context of skiing. *International Journal of Sport Management and Marketing*, *6*(3), 277–291. doi:10.1504/IJSMM.2009.029089

Töglhofer, C., Eigner, F., & Prettenthaler, F. (2011). Impacts of snow conditions on tourism demand in Austrian ski areas. *Climate Research*, *46*(1), 1–14. doi:10.3354/cr00939

Tsiotsou, R. H., & Wirtz, J. (2012). Consumer behavior in a service context. In V. Wells & G. Foxall (Eds.), *Handbook of developments in consumer behaviour* (pp. 147–201). Cheltenham, UK: Edward Elgar.

Vanat, L. (2018). International report on snow & mountain tourism: Overview of the key industry figures for ski resorts (Report No. 10). Retrieved from https://vanat.ch/RM-world-report-2018.pdf.

Won, D., Bang, H., & Shonk, D. (2008). Relative importance of factors involved in choosing a regional ski destination: Influence of consumption situation and recreation specialization. *Journal of Sport & Tourism*, *13*(4), 249–271. doi:10.1080/14775080802577185

Won, D., & Hwang, S. (2009). Factors influencing the college skiers and snowboarders' choice of a ski destination in Korea: A conjoint study. *Managing Leisure*, *14*(1), 17–27. doi:10.1080/13606710802551197

© 2019 The Author(s). This open access article is distributed under a Creative Commons Attribution (CC-BY) 4.0 license.

You are free to:
Share — copy and redistribute the material in any medium or format.
Adapt — remix, transform, and build upon the material for any purpose, even commercially.
The licensor cannot revoke these freedoms as long as you follow the license terms.

Under the following terms:
Attribution — You must give appropriate credit, provide a link to the license, and indicate if changes were made.
You may do so in any reasonable manner, but not in any way that suggests the licensor endorses you or your use.
No additional restrictions

You may not apply legal terms or technological measures that legally restrict others from doing anything the license permits.

***Cogent Social Sciences* (ISSN: 2331-1886) is published by Cogent OA, part of Taylor & Francis Group.**

Publishing with Cogent OA ensures:

- Immediate, universal access to your article on publication
- High visibility and discoverability via the Cogent OA website as well as Taylor & Francis Online
- Download and citation statistics for your article
- Rapid online publication
- Input from, and dialog with, expert editors and editorial boards
- Retention of full copyright of your article
- Guaranteed legacy preservation of your article
- Discounts and waivers for authors in developing regions

Submit your manuscript to a Cogent OA journal at www.CogentOA.com
